# 1058. *In Vitro* and *In Vivo* Antibacterial Activity of Cefiderocol against *Burkholderia* spp

**DOI:** 10.1093/ofid/ofab466.1252

**Published:** 2021-12-04

**Authors:** Merime Oota, Hitomi Hama, Toriko Yoshitomi, Rio Nakamura, Miki Takemura, Yoshinori Yamano, Meredith Hackel, Daniel F Sahm

**Affiliations:** 1 Shionogi & Co., Ltd., Osaka, Osaka, Japan; 2 Shionogi TechnoAdvance Research & Co., Ltd., Toyonaka, Osaka, Japan; 3 IHMA, Inc., Schaumburg, Illinois

## Abstract

**Background:**

*Burkholderia* spp. is an opportunistic pathogen associated with respiratory infections. Cefiderocol (CFDC), a siderophore cephalosporin approved in US and EU, is active *in vitro* against carbapenem-resistant Gram-negative bacteria including *Burkholderia* spp. This study examined *in vitro* and *in vivo* activity of CFDC against *Burkholderia* spp.

**Methods:**

MICs of CFDC and 13 marketed antibacterial drugs against 462 clinical isolates of *Burkholderia* spp. collected in 2014 - 2019 in 13 countries were determined by broth microdilution method according to CLSI guidelines. Only for CFDC, iron-depleted CAMHB was used. In a rat lung infection model, *B. cepacia* ATCC 25416 (CFDC MIC: ≤ 0.031 μg/mL, MEM MIC: 4 μg/mL) was used. Male CD (SD, immunocompetent, n=4-5) rats were infected by intrabronchial inoculation of the bacterial suspension including 1% nutrient agar. The humanized PK in plasma by administration of CFDC 2 g every 8 h (3-h infusion) and MEM 1 g every 8 h (0.5-h infusion) were recreated via the continuous intravenous infusion for 4 days, and the viable cfu in lungs were counted.

**Results:**

Against 462 strains, including 185 MEM non-susceptible isolates, CFDC showed MIC_50_/MIC_90_ of ≤ 0.031/1 µg/mL, which was the lowest among the tested antibiotics. Among 185 MEM non-susceptible isolates, 94% of the isolates exhibited ≤ 4 µg/mL of CFDC MIC. In a rat lung infection model, CFDC and MEM showed bactericidal activity with 2.8 and 2.4 log_10_ CFU/lung decrease compared with non-treated control, respectively. By recreating the humanized PK exposure in this model, 100% and ca.35% of *f*T >MIC of CFDC and MEM in plasma has been achieved, respectively. The bactericidal activities of both compounds vs *B. cepacia* ATCC 25416 would be reasonable because the *f*T >MIC achieved in this model exceeds the target *f*T >MIC (75% for CFDC and 26% for MEM against *Acinetobacter baumannii*, respectively) required to cause 1 log_10_ reduction in murine thigh infection models^1,2)^.

1) M. Sabet. 2019. AAC 2) R. Nakamura. 2019. AAC

In vitro activity of CFDC and comparator agents against Burkholderia spp.

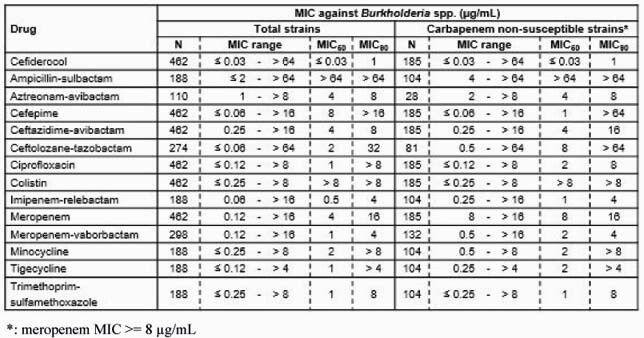

**Conclusion:**

CFDC has potential for treating respiratory tract infections caused by *Burkholderia* spp. In critically ill patients, the recommended dosing regimen achieves 100% of *f*T >MIC of ≤ 4 ug/mL^3)^.3) N. Kawaguchi. 2021. AAC

**Disclosures:**

**Merime Oota, BSc**, **Shionogi TechnoAdvance Research & Co., Ltd.** (Employee) **Toriko Yoshitomi,** -, **Shionogi TechnoAdvance Research & Co., Ltd.** (Employee) **Rio Nakamura, BSc**, **Shionogi TechnoAdvance Research & Co., Ltd.** (Employee) **Miki Takemura, MS**, **SHIONOGI & CO., LTD.** (Employee) **Yoshinori Yamano, PhD**, **Shionogi** (Employee) **Meredith Hackel, PhD MPH**, **IHMA** (Employee)**Pfizer, Inc.** (Independent Contractor) **Daniel F. Sahm, PhD**, **IHMA** (Employee)**Pfizer, Inc.** (Independent Contractor)

